# Cases Intended to Illustrate the Use of the Stethoscope and Percussion in Diagnosis

**Published:** 1827-06

**Authors:** Æneas Mac Andrew

**Affiliations:** Physician to the South London Dispensary.


THE LONDON
### Medical and Physical Journal.

N° 340, VOL. LVII.] JUNE, 1827. [N° 12, *New Series.*

For many fortunate discoveries in medicine, and for the detection of numerous errors, the world is indebted to the rapid circulation of Monthly Journals; and there never existed any work, to which the Faculty, in Europe and America, were under deeper obligations, than to the Medical and Physical Journal of London, now forming a long, but an invaluable, series.—RUSH.

### ORIGINAL PAPERS,
AND
### CASES OBTAINED FROM PUBLIC INSTITUTIONS AND OTHER AUTHENTIC SOURCES.

### DISEASES OF THE CHEST.

*Cases intended to illustrate the Use of the Stethoscope and Percussion in Diagnosis.* By ÆNEAS MAC ANDREW, M.D. Physician to the SOUTH LONDON DISPENSARY.

CASE I. — Redman, a stout married woman, twenty-six years of age, mother of a large family, admitted December 19th, 1826. She has occasionally been liable to fits of taciturnity, lasting for some days, and attended with want of sleep, and the refusal of nourishment. About three weeks ago, she received the intelligence of her father's death; an event that had been concealed from her for some months. She then became silent, refused food, and did not sleep in the night. Five or six days ago, she had cold shivering, followed by heat of skin, pain of head, dyspnœa, cough, and pain of the chest; since which time she has taken some medicines, the nature of which was not ascertained. When visited to-day, she appeared to suffer a great deal; her face was livid; she understood what was said to her, and, without speaking, referred her uneasy sensations to the chest. There was very great dyspnœa, and the respiration was attended with a mucous wheeze, audible to the unassisted ear. On applying the stethoscope, the same wheeze was heard over the whole of the right side of the thorax. Neither the wheeze nor the respiratory murmur could be heard on the left side. Percussion was scarcely employed. She was said to have expectorated some blood in the course of her illness, and two viscid brown coloured sputa were shown. Her pulse was quick, feeble, and very easily compressed; tongue clean; bowels confined for three days; involuntary discharge of urine. She died in six hours.

The body was examined thirty hours after death. Great embonpoint. Percussion of the chest afforded no symptom whatever: the sound was equally dull on both sides. There were two inches of fat under the integuments of the abdomen, and somewhat less on the thorax. The left side of the thorax contained about two pints of a dirty brown-coloured fluid, having whitish flocculi suspended in it, and being perfectly inodorous. The lung on the same side was much diminished in size, and pressed towards the spine. It adhered to the diaphragm by means of coagulable lymph, which was very easily torn through. The pleura was much more vascular than natural, and in many places was covered with lymph. The lung was thin and flaccid, and of a livid colour: it did not crepitate; it sunk in water. When cut into, it had a dark red colour, interspersed with greyish-coloured streaks. One portion of it was softer than the rest. It was not attempted to inflate it, no blowpipe being at hand. The right lung was perfectly healthy. The bronchi contained a reddish-coloured mucus, but their inner membrane was not preternaturally red. Heart without disease. The abdominal viscera appeared healthy on a superficial examination. Head not examined.

This patient appears to have been affected, in the first instance, with melancholia; on which supervened an attack of pleuritis, that proved fatal. When first visited, she was evidently in the agony, which circumstance prevented a very minute examination. The history obtained from her friends was enough to render an acute inflammation of the respiratory organs extremely probable; and the additional information afforded by the stethoscope was, that the respiration was carried on in the right lung alone, and consequently that the disease was situated on the left side. It also showed that the right lung was so gorged with mucous fluid, as to render an early death inevitable. It was scarcely possible to determine whether the lung or the pleura was most affected: the severe pain of the side indicated the former, and the appearance of the sputa the latter. Percussion was of no use in this patient, for the accumulation of fat was so great as to prevent any hollow sound being heard. This general want of sonorosity, however, only deprives us of a symptom, but does not lead to any erroneous conclusion. The condition of the left lung is a good example of the manner in which the appearance of the pulmonic tissue may be altered by mere pressure, without any change in its structure.

Case II.—William Coldford, a sailor, of muscular habit and short stature, supposed to be about thirty years of age; admitted March 25th, 1826. He states that, about fifteen months ago, whilst using strong exertion in pulling a rope, he was attacked with pain of chest and difficulty of breathing, for which a blister was applied. These complaints have never entirely left him since that time, and have been accompanied with palpitations, bloody expectoration, and occasional œdema; symptoms that have always been aggravated by exposure to cold.

He complains to-day of great dyspnœa, so as to be unable to lie down in bed. There is pain under the sternum, coming on occasionally, and augmented by coughing. He expectorates a frothy viscid mucus, slightly tinged with blood. The chest, throughout the whole of its extent, yields a dull sound on percussion. On applying the stethoscope, the respiratory murmur may be heard in almost every point of the chest; it appears to be somewhat weaker on the left side posteriorly. The impulse communicated by the pulsations of the heart is strong, particularly towards the sternum, and each contraction of the ventricles is attended with a loud "*bruit soufflet*." The pulse in the left arm is strong and hard; in the right arm it is less hard, and more easily compressible. Tongue whitish; appetite tolerably good; bowels open; urine high coloured; slight œdema of the feet; the face is pale and swollen, and the conjunctiva has a yellow tinge, but his friends assert that this is his natural appearance. For seven days he has been taking a mixture containing Digitalis and Conium.

V.S. ad ℥ xij.—Hydrarg. Submur. gr. v.; Pulv. Jalapæ gr. xxx. M. fiat pulvis statim sumendus.—Very spare diet.

26th.—Dyspnœa somewhat relieved; pain less severe; cough less frequent. The bowels have been freely opened. The blood drawn is firmly coagulated, but without any buffy coat.

27th.—No change.

Sumat Tinct. Digitalis m. x. bis die.—An oily emulsion for his cough.

28th.—Complains much of pain under the sternum, which however is not constant. The pulse is less strong; some degree of nausea. Œdema of the feet increased.

Admoveantur sterno Hirudines x.—Intermittantur Tinct. Digitalis et Mist. Oleosa.

29th.—Leeches bled well; pain of chest relieved. He passed a very restless night. Skin and eyes are still of a yellow tinge; and he complains of pain on pressure in the hepatic region. Œdema diminished.

Rep$^{r}$ Tinct. Digitalis.

30th.—Pain of chest has returned; œdema is increased. *Bruit soufflet* may be heard in the chest as formerly, and also for some inches below the pit of the stomach, along the course of the abdominal aorta. Abdomen painful on pressure.

V.S. ad ℥ x.—Mistura Mucilaginosa pro tussi.

31st.—Pain of chest relieved. Blood not buffy. Œdema as before.

℞. Acet. Potassæ ʒ iv.; Aquæ Menth. Pip. f. ℥ x. fiat mistura cujus capiat cochlear. ij. magna ter die.—Misceatur Supertart. Potassæ cum potu communi ad gratum acorem efficiendum.

April 1st.—Pain of chest and dyspnœa are severe.

Hirudines xx. sterno.

2d.—Pain gone. Dyspnœa extreme. Pulse tolerably strong. He died in the evening, when standing with his breast leaning on a chair, the posture that always gave him most relief.

On examining the body, there was found to be great œdema of the lower extremities, and much adipose substance under the integuments of the thorax and abdomen.

*Chest:* Pericardium contains about five ounces of fluid. Heart so much enlarged as to be nearly twice the size of the subject's fist. Parietes of left ventricle considerably thickened; but of the right, scarcely at all. Semilunar valves of the aorta considerably thickened at their central margin, by a deposition of cartilaginous matter. Coagula of blood in both ventricles. Pulmonary artery sound. Right lung, throughout its whole extent, adhering to the pleura, from which it cannot be separated without using force, particularly towards the diaphragm. Left lung also adhering to the pleura, but less extensively, and more easily separated. The lungs crepitate on pressure in every direction; lower lobe of right lung partially changed, so as to have a yellow colour. The trachea and larger bronchial tubes contain much frothy mucus, and their lining membrane is of a vivid red colour. Frothy mucus issues abundantly from incisions made into the lungs.

*Abdomen:* Smaller intestines distended with air. Inner coat of the stomach somewhat reddened. Portions of the mucous membrane of the jejunum are elevated from the effusion of air, and the same emphysematous swelling extends into the adjacent mesentery. Liver somewhat enlarged and hardened.

*Head* not examined.

The age, constitution, and habits of this patient rendered him particularly obnoxious to those affections of the heart that depend upon a morbid increase of strength. The attack of pleurisy, from which he dated his complaints, appears to have been severe, and inadequately treated. This disease gave rise to the firm adhesions by which the right lung was fixed to the side of the chest in its whole extent, and, by preventing the natural sliding of the lung on the parietes of the chest during inspiration, it gave rise to a certain degree of dyspnœa. This alone might cause enlargement of the heart in a person predisposed to it; but in this patient there existed at the same time a change in the structure of the aortic valves, which is a more common and less equivocal cause of cardiac enlargement. This enlargement was marked by symptoms perhaps sufficiently characteristic, without having recourse to the stethoscope; which, however, besides rendering more evident the great increase in the force of the ventricular contractions, allowed us to advance a link in the chain of morbid actions, and nearly proved the existence of an obstruction at the root of the aorta. It is true that the *bruit soufflet* may often be heard, without being caused by any change of structure, and, when taken alone, it is a very equivocal symptom; yet when, as in the present case, it is never absent, is very distinct, and attended with other marks of diseased heart, the existence of some obstruction to the circulation at the part where it is loudest can scarcely be doubted.

The difference of the pulse in the two arms does not appear to have been at all connected with the disease of the heart. It most probably arose from a congenital variation of size in the radial arteries, which perhaps would be found to exist more frequently, if oftener sought after. Most authors recommend the pulse being felt in both arms, but the rule is generally neglected, and it is manifest that this may lead to errors in practice.

The bleeding, purging, and digitalis, were prescribed with the view of diminishing the quantity of circulating fluid, of lessening the excess of strength in the action of the heart, and of cutting short the bronchial inflammation. The diuretics were afterwards employed to carry off the anasarcous swelling; but the patient died suddenly, as frequently happens in disease of the heart.

The effusion of air under the mucous tissue of the jejunum, and between the laminæ of the mesentery, was an appearance new to me; nor have I been able to meet with any account of it amongst authors.

Case III.—James Nowlans, aged forty-five, a horner, admitted 29th January, 1827. On February 1st, the following history of his complaints was obtained: About seven months ago, he was attacked with pain of the right side of the thorax, cough, and dyspnœa. The cough has never entirely left him since that time; he has expectorated much, and become very thin and weak. He has been confined to his bed for a month.

When examined to-day, he was found to be extremely emaciated. He complains much of dyspnœa, and coughs almost incessantly. The inspirations are twenty-eight in a minute. The expectorated matter is not very abundant: it consists of mucus, tinged with blood; is of a uniform red colour; and adheres to the vessel, hanging from it when inverted, in one continuous mass. He has little pain of chest. On employing percussion at the superior and anterior parts of the chest, the sound on the right side is found to be hollow. On the left side, the sound is distinctly stronger. Little difference is perceptible on other parts of the chest. On applying the stethoscope, the respiratory murmur may be heard over the whole of the right side, mixed with wheezes, and partially obscured by them: on the superior parts, the wheeze is mucous; on the inferior, sonorous. No respiratory murmur can be heard on the left side of the thorax, where the sound is so strong on percussion; on other parts of the left side it is weak, and occasionally mixed with a sonorous wheeze. Although he breathes more easily when sitting up, he lies on his back from debility; if on either side, the dyspnœa is augmented. Pulse 100, easily compressed; tongue whitish; appetite gone, he can take nothing but milk and water; bowels open once a-day. Two days ago, a blister was applied to his chest, but did not rise well. He has taken for two nights a pill composed of Conium and Ipecacuan.

Emplast. Lyttæ amplum dexteriori lateri thoracis. Capiat Tinct. Digitalis m. v. quater indies.—℞. Extract. Conii ʒj.; Extract. Hyoscyam. ʒss.; Mucilag. Acac. f.℥ iv. M. sumat f.℥ ss. ter die.

Feb. 2d.—He has scarcely slept at all, on account of pain from the blister, which has risen very well, but been applied to the left side. The dyspnœa is not so urgent; the cough is less troublesome; the sputa are diminished in quantity, but have the same character as yesterday. The stethoscope, as far as it can be applied, gives the same results, only that, on the superior and anterior part of the left side, a sonorous wheeze is heard. It is evident, however, that the wheeze is not produced on the left side, but on the right, where it is very strong. Pulse 102; tongue dry. Three stools.

Contr omnia.

3d.—He has taken, contrary to orders, some decoction of Sassafras, and a glass of port-wine. He slept better. Inspirations are twenty-six in a minute. The sputa are more deeply tinged with red. Percussion and mediate auscultation afford the same results as on the first. Three very scanty stools.

Continue.

4th.—He passed the night tolerably well, not appearing worse than yesterday. At seven this morning, the dyspnœa becoming urgent, he was much agitated, declared that he had burst a blood-vessel; and, having coughed up some scarlet-coloured frothy blood, he expired.

Permission to examine the body was not granted till the end of three days, when putrefaction had made considerable progress; the colour of the integuments being greenish, the cuticle easily detached, and the odour strong. The body was much emaciated. There was no difference of size in the two sides of the thorax, nor did they yield a different sound on percussion. A puncture made on the superior and anterior part of the left side allowed no air nor fluid to escape; but, on dividing the cartilages of the sixth and seventh ribs on the same side, a quantity of air rushed out with some force, so as to be distinctly audible. On both sides the lungs adhered to the pleura for a small extent, and were easily detached; each side contained about half a pint of bloody serum.

On the superior lobe of the lung were observed two foramina, at the distance of an inch and a half from each other, and large enough to admit a goosequill: they were apparently lined with pleura, and terminated in a cul-de-sac, so as to be probably an original variation in structure. The trachea, the large blood-vessels below the atlantal margin of the sternum, and the œsophagus, were now cut through, in order that the lungs might be removed from the thorax, and examined with greater ease. This being done incautiously, an aneurismal sac was cut into, and part of it detached with the lungs.

The bodies of the third, fourth, and fifth vertebræ were completely denuded, and partially absorbed. They all felt rough to the touch, but more particularly the fourth. The intervertebral substances projected considerably beyond the vertebræ.

On slitting up the aorta, the inner membrane was found much reddened; and, about half an inch below the origin of the left subclavian artery, there was observed a circular opening, with a well-defined smooth margin, and nearly an inch in diameter. This led into the aneurismal sac, which was about the size of a hen's egg, and contained coagula of a fibrinous nature, and easily separable into layers. On their removal, a communication was found to exist between the sac and the left bronchial tube, immediately below the bifurcation of the trachea. The lining membrane of the trachea was of a dark red colour, and the left bronchial contained portions of dark coagulated blood. The left lung was of a dark colour throughout; it crepitated, and swam in water. On pressing it firmly, a thin bloody fluid exuded abundantly, together with portions of coagulated blood. The right lung was not so dark coloured, but also yielded a bloody fluid on pressure. The pericardium contained about two ounces of bloody fluid. The heart was pale, flaccid, and nearly empty of blood.

In the abdomen, the intestines were distended with flatus. The mucous coat of the stomach of a bright red colour towards its cardiac orifice. The smaller and larger intestines healthy. The renal veins and vena cava contained no blood, but were distended with air, which escaped on a puncture being made. The liver adhering to the diaphragm in the whole of its convex surface: it was easily torn, and had a dirty dark green colour. The spleen was still farther advanced in putrefactive dissolution.

The head was not examined.

This patient laboured under three diseases of the chest, which had no necessary connexion with each other. The aneurism, which was the most serious affection, and the immediate cause of his death, afforded no symptom by which it could have been detected during life, and scarcely any that could have led to a suspicion of its existence. Neither in the history of the disease nor in the examination of the patient, did any circumstance occur which could not be accounted for by the presence of long-continued inflammation of the bronchial membrane, and by the pneumo-thorax. The sac had attained a considerable size, without any remarkable derangement of the circulation; the osseous bodies of the vertebræ had been partially absorbed, without any local pain or external tumor; and the bronchial tube had yielded to the same process, without a dyspnœa greater than what accompanies many other diseases of the chest.

Aneurism of the aorta, in its early stages, affords us no characteristic symptom; and, in the majority of cases, it is as much concealed from the explorator by the stethoscope as from any other diligent observer. Pneumothorax is an affection that can scarcely be discovered without the assistance of percussion and mediate auscultation, and these methods alone suffice to demonstrate its presence. They must, however, be taken in conjunction, so as to correct each other. In this patient, the tympanitic sound in the part where the air was effused was very remarkable; and the total want of respiration here contrasted strongly with the noisy breathing on the other side of the chest. On one occasion, the wheeze was distinctly heard on the left side; but a little attention showed that this was merely the extension of the wheeze which was very loud on the right side. On opening the body, the air was found to have changed its situation; but this might easily have happened, either during the struggles of the agony, or by moving the body after death.

The cause of the air being formed in the pleural cavity in this patient, is not very evident. There was no communication between the bronchi and the pleura; neither can we suppose that gas had been evolved from the decomposition of the pleuritic effusion; for the quantity of fluid was but small, it was not evidently changed by putrefaction, and it was precisely the same in both sides of the chest, although the air had only been formed in one. Succussion, too, had been tried during life, but afforded no result. However this may be, its existence was evident, and it rendered the prognosis more unfavourable, without leading to any change of treatment.

The inflammation of the bronchial membrane was, perhaps, sufficiently marked by the constant cough,—the great dysnœa without much pain,—the appearance of the sputa,—and the frequency of the pulse; but the mucous and sonorous wheezes were more certain indications of its presence. It may be questioned whether the sanguineous appearance of the sputa depended upon the increased action of the vessels of the mucous membrane, or if it were caused by the escape of a minute portion of blood from the aneurismal sac. The former opinion is by far the more probable. We can scarcely doubt that, whenever the communication between the sac and the bronchial tube was effected, the blood was poured out in such quantity as to prove quickly fatal, by producing either suffocation or syncope.

161, *Great Surrey-street;*

*March* 20, 1827.

